# Psychometric properties of the Harmony in Life Scale in South African and Ghanaian samples

**DOI:** 10.4102/ajopa.v5i0.122

**Published:** 2023-02-28

**Authors:** Amanda Cromhout, Lusilda Schutte, Marié P. Wissing, Angelina Wilson Fadiji, Tharina Guse, Sonia Mbowa

**Affiliations:** 1 Africa Unit for Transdisciplinary Health Research (AUTHeR), Faculty of Health Sciences, North-West University, Potchefstroom, South Africa; 2 Department of Educational Psychology, Faculty of Education, University of Pretoria, Pretoria, South Africa; 3 Department of Psychology, Faculty of Humanities, University of Pretoria, Pretoria, South Africa; 4 Centre for Social Development in Africa, Faculty of Humanities, University of Johannesburg, Johannesburg, South Africa

**Keywords:** harmony in life, South Africa, Ghana, validity, reliability, measurement invariance

## Abstract

Harmony is regarded as important for well-being in many cultures. However, (cultural) differences in the meanings and manifestations of harmony may impact the equivalence of measures of harmony in life, as well as the associations between harmony and other well-being constructs. This study aimed to investigate the factorial, convergent and divergent validity, and measurement invariance of the Harmony in Life Scale (HILS) in South African and Ghanaian samples. Confirmatory factor analysis was applied to data from three South African samples (two multicultural samples completed the HILS in English; and a Setswana-speaking sample completed the HILS in Setswana) and one Ghanaian sample (completed the HILS in English). Sample sizes ranged between *n* = 400 and *n* = 427. Good fit indices were obtained for all samples, except for the Setswana-speaking sample from South Africa. In all instances the HILS showed good internal consistency reliability and convergent and divergent validity. Full scalar invariance was supported for the two multicultural South African samples, but only partial scalar invariance when data from the Ghanaian sample were added to the analysis. The HILS shows potential for future use in all samples, except the Setswana-speaking sample. Findings emphasise the importance of considering cultural and/or contextual and linguistic differences and how these may influence the measurement of psychological constructs. Future research should qualitatively explore the meanings and manifestations of harmony in various African and other global contexts in local languages.

**Contribution:**

This study is the first to investigate the psychometric properties of the original English version of the HILS in South African and Ghanaian samples, as well as a Setswana translation of the scale. The study contributes to the understanding of harmony in life and the measurement thereof in diverse contexts, in this case specifically focused on African samples, and may, in turn, inform interventions and evaluation of interventions.

## Introduction

Harmony in life is associated with well-being and quality of life and seems to be valued across cultures. In a multi-country study by Delle-Fave et al. (2011), participants were asked what happiness is to them and, in the domain of psychological definitions of happiness (other domains included family, work, health, etc.), 25.4% of the responses included reference to harmony or balance, which was higher than for any other subcategory in the psychological definitions domain. Delle-Fave et al. (2016) found that almost 30% of the definitions of happiness in the psychological definitions domain referred to inner harmony, while 29.11% of the responses referred to balance. Other research supports the notion that harmony or balance is associated with well-being (e.g. Delle-Fave et al., 2022; Di Fabio & Tsuda, 2018; Lomas, 2021; Lomas et al., 2022; Schutte et al., 2022; Sirgy, 2019). Although most cultures seem to value harmony as an important aspect of life and well-being, the construct may have different nuances and manifestations in different cultural groups. Factors such as differences in language and culture can impact the equivalence of measures of harmony. Furthermore, depending on cultural meanings of harmony, the construct may have varying associations with other well-being constructs across cultures. Therefore, in order to promote well-being cross-culturally, it is important to understand how the meanings, manifestations and measurement of harmony overlap and differ across groups, especially in under-researched African contexts.

### Conceptualisation of harmony

Harmony has been described as ‘a global and overall assessment of whether one’s life involves balance, mindful non-judgmental acceptance, fitting in and being attuned with one’s life’ (Garcia et al., 2014, p. 5). According to the Meriam-Webster dictionary, harmony refers to ‘a pleasing arrangement of parts’ or congruence, as well as agreement and accord, and internal calm or tranquillity (https://www.merriam-webster.com/dictionary/harmony). In Eastern philosophy, harmony suggests a favourable relation among different things that exist (Li, 2006), while harmony is understood in terms of social relationships in African contexts (see Metz, 2017 for a discussion). Describing harmony from a psychological well-being perspective, Kjell et al. (2016) proposed that harmony ‘encourages a holistic world view that incorporates a balanced and flexible approach to personal well-being that takes into account social and environmental contexts’ (p. 894). Applying latent semantic analysis, Kjell et al. (2016) found that participants associated harmony with balance, accord, agreement, concord and tranquillity, and that these linked to concepts that tap into selflessness, interconnectedness and interdependence.

Considering these links with selflessness, interconnectedness and interdependence, one may expect that harmony will be especially important in cultures with an interdependent self-construal (e.g. African, Asian, Latin-American and Southern European cultures) where individuals are viewed as more connected, rather than differentiated from others, compared to cultures with an independent self-construal (e.g. North American and many Western European cultures), where individuals view themselves as autonomous and independent from others (Kitayama et al., 2020; Markus & Kitayama, 1991).

In a study investigating cross-cultural variations and similarities of happiness and subjective well-being, Uchida et al. (2004) argued that, although happiness is likely to be universal, the experience thereof is embedded in socio-cultural contexts. Happiness is therefore encompassed in rich, associative networks that vary cross-culturally. This same argument can apply to the conceptualisation and experience of harmony and other facets of well-being across cultures. Considering that culture consists of distinct sets of values, attitudes and behaviours that form value schemas or value orientations (Connor & Becker, 2003, 2006; Rokeach, 1973, 1979), it can be expected that these differences can influence how constructs are interpreted (thus influencing the meaning attached to the construct) and manifest across cultures which, in turn, influence the measurement of the construct. It is therefore important that the validity of measuring instruments assessing harmony in life is investigated for different cultural groups.

### Measuring harmony in life: The Harmony in Life Scale

The Harmony in Life Scale (HILS, Kjell et al., 2016) is a 5-item measure of overall harmony in life. Kjell et al. (2016) proposed that harmony in life is complementary to satisfaction with life in explaining the cognitive component of subjective well-being as measured by the Satisfaction with Life Scale (SWLS, Diener, 1984; Diener et al., 1985; Kjell et al., 2016). They argued that the cognitive aspects of psychological functioning relevant when evaluating harmony in life, stand in contrast with the evaluations relevant to satisfaction with life (see Kjell et al., 2016, for a discussion of this aspect). More specifically, evaluations in the HILS involve psychological balance and flexibility in life, whereas evaluations in the SWLS involve comparing actual life circumstances to expected life circumstances (Kjell et al., 2016).

Kjell et al. (2016) reported a Cronbach’s alpha value of 0.90 for the HILS, sufficient test-retest reliability (*r* = 0.77), and support for convergent and discriminant validity. In a validation study of the Turkish translation of the HILS, Satici and Tekin (2017) found support for a unidimensional structure, as well as for convergent and discriminant validity of the scale. Cronbach’s alpha values ranged from 0.77 to 0.79, composite reliability scores from 0.78 to 0.80, and test-retest reliability was supported. Singh et al. (2016) also found support for a unidimensional structure of the Hindi translation of the HILS and reported a Cronbach’s alpha value of 0.88.

### Harmony and well-being

If the meaning of harmony may differ cross-culturally, the underlying motives for harmony, as well as the predictors of harmony, may also vary across cultures (cf. Uchida et al., 2004). There may therefore be variation in how harmony associates with other indicators of well-being depending on the cultural context.

Studies that investigated associations between harmony in life and other well-being indicators include the original validation study by Kjell et al. (2016) where the HILS showed positive correlations with satisfaction with life (*r* = 0.74), subjective happiness (*r* = 0.71), facets of psychological well-being such as environmental mastery (*r* = 0.64), personal growth (*r* = 0.25), positive relations (*r* = 0.43), and self-acceptance (*r* = 0.65) but did not correlate with autonomy (*r* = 0.03), and purpose in life (*r* = 0.08). The scale was correlated negatively with measures of depression (*r* = -0.39), anxiety (*r* = -0.13) and stress (*r* = -0.26; Kjell et al., 2016) in a mainly American sample (*n* = 476, United States [US] = 406, India = 37, and other countries = 33).

In a validation study of the Turkish translation of the HILS, Satici and Tekin (2017) found that the HILS correlated positively with life satisfaction (*r* = 0.44), positive affect (*r* = 0.35), subjective happiness (*r* = 0.43), and subjective well-being (*r* = 0.51), and negatively with negative affect (*r* = -0.31). Harmony in life was positively predicted by flourishing (ß = 0.55) and negatively predicted by depression (ß = -0.50), anxiety (ß = -0.40), and stress (ß = -0.37) in a sample of Turkish university students (*n* = 253).

### The present study

Despite the importance of harmony for people’s well-being (cf. Delle-Fave et al., 2011), research on harmony is sparse, also in African contexts. Considering that the meanings, manifestations and the measurement of harmony in life seem to be informed by cultural values and judgements (e.g. Satici & Tekin, 2017), this study aims to investigate the factorial, convergent and divergent validity and measurement invariance of the HILS (Kjell et al., 2016) in South African and Ghanaian samples. The associations between the HILS and selected measures of well-being and ill-being were also examined. Some of the scales used in the current study measure similar constructs to the scales used in the validation studies by Kjell et al. (2016) and Satici and Tekin (2017), which enabled us to see how findings in African contexts compare to the findings of other studies where samples from other cultural groups were used. South Africa and Ghana were selected because research teams who conduct research in these two countries have established collaboration relationships and administered the HILS in their studies. While this selection is not representative of the wider African population, the countries present geographical diversity, with South Africa being in Southern Africa and Ghana in West Africa.

Validation studies on the HILS are still limited, and include the studies of Kjell et al. (2016, English version), Kjell and Diener (2021, English version), Satici and Tekin (2017, Turkish translation), and Singh et al. (2016, Hindi translation). As far as we could establish when searching in the literature, this study is the first to investigate the psychometric properties of the original English version of the HILS in South African and Ghanaian samples, as well as a Setswana translation of the scale. The study contributes to the understanding of harmony in life and the measurement thereof in diverse contexts, in this case specifically focused on African samples, and may, in turn, inform interventions and evaluation of interventions.

## Methods

### Participants

Data from four nonprobability samples gathered in different studies were used: Samples 1 and 2 were multicultural adult South African samples, Sample 3 consisted of Setswana-speaking adults from South Africa, and Sample 4 was an African adult sample from Ghana. See Table 1 for a description of the respective samples. Samples 1, 2 and 4 completed the research battery in English, and Sample 3 in Setswana. For all samples, participants had to be 18 years or older to participate in the study. In addition, for Samples 1 and 4 participants also had to have at least a Grade 12 level of education and be proficient in English because participants had to complete the research battery in English. A Grade 12 level of education was assumed to indicate sufficient English proficiency to complete the research battery. For Sample 2, participants had to be able to read and understand English and had to have access to the online survey through a computer or mobile device. For Sample 1, the data formed part of the FORT 3 research project (FORT = Fortology Project [forté=strength]); with the FORT 3 subproject applicable for this study named: ‘The prevalence of levels of psychosocial health: Dynamics and relationships with biomarkers of (ill)health in South African social contexts (Wissing, 2008/2012); and for Sample 2, the International Hope Barometer Programme (Krafft et al., 2018). For Sample 3, data formed part of the mental health leg of the 2017–2019 round of data gathering in the longitudinal, multidisciplinary Prospective Urban and Rural Epidemiology – South Africa (PURE-SA) study (Teo et al., 2009), North West province, which involved an overlap between PURE-SA and the FORT 3 research project. For Sample 4, data formed part of the Ghana leg of the Eudaimonic and Hedonic Happiness Investigation (EHHI; Delle-Fave et al., 2011, 2016; Wilson, 2017), although additional items outside the EHHI were added.

**TABLE 1:** Description of Samples 1 to 4.

Variable	Sample 1			Sample 2			Sample 3			Sample 4		
	Multicultural, SA1	Mean	SD	Multicultural, SA2	Mean	SD	Setswana, SA	Mean	SD	Ghana	Mean	SD
*n*	287			427			400			420		
**Age**	-	36.72	13.25	-	38.87	14.50	-	61.57	8.87	-	41.37	9.42
**Gender (%)**												
Male	33.8	-	-	30.4	-	-	29.0	-	-	42.4	-	-
Female	64.8	-	-	69.6	-	-	70.5	-	-	55.0	-	-
Missing	1.4	-	-	-	-	-	0.5	-	-	2.6	-	-
**Home language (%)**												
English	25.8	-	-	-	-	-	–	-	-	-	-	-
Setswana	4.5	-	-	-	-	-	82.5	-	-	-	-	-
Afrikaans	47.4	-	-	-	-	-	–	-	-	-	-	-
Other	18.8	-	-	-	-	-	17.3	-	-	-	-	-
Missing	3.5	-	-	-	-	-	0.3	-	-	-	-	-
**Population group**												
Black people	-	-	-	34.0	-	-	-	-	-	-	-	-
Mixed race people	-	-	-	2.1	-	-	-	-	-	-	-	-
Indian people	-	-	-	4.7	-	-	-	-	-	-	-	-
White people	-	-	-	57.8	-	-	-	-	-	-	-	-
Other	-	-	-	1.4	-	-	-	-	-	-	-	-
**Level of education (%)**												
Secondary	18.1	-	-	-	-	-	-	-	-	49.3	-	-
Tertiary	22.4	-	-	-	-	-	-	-	-	40.2	-	-
Post-graduate	45.3	-	-	-	-	-	-	-	-	6.9	-	-
Missing	3.1	-	-	-	-	-	-	-	-	3.6	-	-
Did not finish school	-	-	-	0.5	-	-	-	-	-	-	-	-
High school up to Grade 10	-	-	-	1.6	-	-	-	-	-	-	-	-
High school up to Grade 12	-	-	-	18.3	-	-	-	-	-	-	-	-
Diploma	-	-	-	11.7	-	-	-	-	-	-	-	-
University degree	-	-	-	67.9	-	-	-	-	-	-	-	-
No formal schooling	-	-	-	-	-	-	22.1	-	-	-	-	-
Gr. 1–3	-	-	-	-	-	-	13.5	-	-	-	-	-
Gr. 4–7	-	-	-	-	-	-	34.0	-	-	-	-	-
Gr. 8–9	-	-	-	-	-	-	17.3	-	-	-	-	-
Gr. 10–12	-	-	-	-	-	-	12.9	-	-	-	-	-
Missing	-	-	-	-	-	-	0.3	-	-	-	-	-
**Standard of living†**												
Below average	2.1	-	-	-	-	-	-	-	-	8.6	-	-
Average	66.6	-	-	-	-	-	-	-	-	60.7	-	-
Above average	28.9	-	-	-	-	-	-	-	-	10.7	-	-
Missing	2.4	-	-	-	-	-	-	-	-	-	-	-

Note: ‘-’, = The question or response option did not form part of the questionnaire.

SD, standard deviation; Gr., grade; SA, South African; SA1, South African Sample 1; SA2, South African Sample 2.

† , Based on participants’ subjective rating of their standard of living.

### Measures

Different scales were included in the research battery used for each of the samples. The following selected scales are relevant for this study.

#### Socio-demographic questionnaire

Socio-demographic data on variables such as gender, age, home language, population group, level of education and standard of living were collected across the samples.

#### Harmony in Life Scale

The Harmony in Life Scale (HILS, Kjell et al., 2016) comprises a single scale (no subscales distinguished) with five items, and measures individuals’ subjective perception of the overall harmony in their life on a 7-point Likert-type scale that ranges from 1 (*strongly disagree*) to 7 (*strongly agree*). Detail on previous findings pertaining to the scale’s psychometric properties was presented in the ‘Introduction’ section.

#### The Satisfaction with Life Scale

The Satisfaction with Life Scale (SWLS, Diener et al., 1985) measures the global judgement of satisfaction with one’s life as a whole through five items on a 7-point Likert-type scale ranging from 1 (*strongly disagree*) to 7 (*strongly agree*). Diener et al. (1985) reported sufficient internal consistency reliability (α = 0.87) and a test-retest reliability score of 0.82. Wissing and Van Eeden (2002) found support for the unidimensional structure of the SWLS in South African samples, and reported Cronbach’s alpha values of 0.70, 0.83 and 0.85 for young adults (ages 18–35), middle adults (ages 36–64) and older adults (ages 65 and older), respectively. Appiah et al. (2020) reported a unidimensional structure with the residuals of items 4 and 5 correlated, and sufficient internal consistency reliability with ɷ = 0.87 for the Twi-translation of the scale in a rural adult Ghanaian sample.

#### The Mental Health Continuum – Short Form

The 14-item Mental Health Continuum – Short Form (MHC-SF; Keyes et al., 2008) comprises three subscales, namely Emotional Well-being (MHC_EWB), Social Well-being (MHC_SWB) and Psychological Well-being (MHC_PWB). The scale measures positive mental health on a 6-point Likert-type scale that ranges from 0 (*never*) to 5 (*every day*). Lamers et al. (2011) reported a three-factor solution with Cronbach’s alpha values of 0.89 (MHC-SF total), 0.83 (MHC_EWB), 0.74 (MHC_SWB) and 0.83 (MHC_PWB) in a Dutch sample between the ages of 18 and 87 years. In South Africa, Keyes et al. (2008) found marginal support for a three-factor solution and reported Cronbach’s alpha values of 0.74 (total MHC-SF), 0.73 (MHC_EWB), 0.59 (MHC_SWB) and 0.67 (MHC_PWB) in a Setswana-speaking sample. In another South African study, Schutte and Wissing (2017) found support for a three-factor bifactor exploratory structural equation modelling model (with item 5 removed), and reported sufficient model-based omega coefficients of composite reliability for the global positive mental health factor in English (ω = 0.88), Afrikaans (ω = 0.90) and Setswana (ω = 0.86) student samples. Appiah et al. (2022) found that a bifactor exploratory structural equation modelling model best fitted the data in a rural adult Ghanaian sample who completed the Twi version of the MHC-SF. Omega reliability coefficients were high (ω = 0.97) for the general positive mental health factor, marginal for MHC_EWB (ω = 0.51) and MHC_SWB (ω = 0.57), and low for MHC_PWB (ω = 0.41).

#### The Meaning in Life Questionnaire

The Meaning in Life Questionnaire (MLQ; Steger et al., 2006) comprises 10 items that measure meaning in life by means of two 5-item subscales, namely Presence of Meaning (MLQ_P; measuring the subjective experience of the meaningfulness of one’s life) and Search for Meaning (MLQ_S; measuring the motivation to find meaning or to better understand the meaning of one’s life) on a Likert-type scale ranging from 1 (*absolutely untrue*) to 7 (*absolutely true*). Steger et al. (2006) reported a 2-factor structure with sufficient Cronbach’s alpha reliability scores for the MLQ_P (between 0.82 and 0.86) and the MLQ_S (between 0.86 and 0.87) in American student samples. In South Africa, Temane et al. (2014) found support for a two-factor structure when the English version was completed by a multicultural student group. They also reported sufficient reliability with Cronbach’s alpha values of 0.85 (MLQ_P) and 0.84 (MLQ_S).

#### The Affectometer-2

The Affectometer-2 (AFM-2; Kammann & Flett, 1983) is the abbreviated version of the AFM 1 and comprises 20 items that measure general happiness or sense of well-being on a scale with five response options (1 = *not at all*; 2 = *occasionally*; 3 = *some of the time*; 4 = *often*; 5 = *all of the time*). The scale has two subscales, namely Positive Affect (AFM2_PA) and Negative Affect (AFM2_NA). Kammann and Flett (1983) reported Cronbach’s alpha reliability scores of 0.88 (AFM2_PA) and 0.93 (AFM2_NA). In South Africa, Wissing et al. (2008) reported Cronbach’s alpha scores of 0.64 (AFM2_PA) and 0.79 (AFM2_NA) for the English version of the scale in a Setswana-speaking sample. Appiah et al. (2020) found support for a two-factor bifactor exploratory structural equation modelling model and reported reliability scores of ɷ = 0.88 (AFM2_total), ɷ = 0.43 (AFM2_PA) and ɷ = 0.72 (AFM2_NA) for the Twi-translation of the scale in a rural adult Ghanaian sample.

#### The Positive Affect and Negative Affect Schedule

The Positive Affect and Negative Affect Schedule (PANAS; Watson et al., 1988) comprises 20 items, and measures positive affect (PANAS_PA, 10 items) and negative affect (PANAS_NA, 10 items), respectively. Respondents must indicate the extent to which they experienced different positive and negative emotions over a certain period of time on a scale with five response options (1 = *very slightly or not at all*, 2 = *a little*, 3 = *moderately*, 4 = *quite a bit*, 5 = *extremely*). Watson et al. (1988) reported a 2-factor solution with Cronbach’s alpha values above 0.80. Only the PANAS_NE is relevant to this study.

#### The Scale of Positive and Negative Experiences

The Scale of Positive and Negative Experiences (SPANE; Diener et al., 2010) comprises 12 items and measures general positive experiences (SPANE_PE, 6 items) and negative experiences (SPANE_NE, 6 items), based on how often the respondent experienced the feelings during a 4-week period. The scale is in Likert-format, and ranges from 1 (*very rarely or never*) to 5 (*very often or always*). Separate scores are calculated for positive and negative experiences, respectively, and/or a balance score by subtracting the score for negative experiences from the score for positive experiences. Diener et al. (2010) reported Cronbach’s alpha values of 0.87 (SPANE_PE), 0.81 (SPANE_NE) and 0.89 (SPANE_balanced) and good convergent validity with measures of emotion, life satisfaction, well-being and happiness. In South Africa, Du Plessis and Guse (2017) reported Cronbach’s alpha values of 0.84 (SPANE_PE), 0.79 (SPANE_NE) and 0.85 (SPANE_balanced) in a multicultural student sample, with the SPANE_PE correlating positively with well-being and life satisfaction, and the SPANE_NE correlating negatively with well-being and life satisfaction.

#### The Subjective Happiness Scale

The Subjective Happiness Scale (SHS; Lyubomirsky & Lepper, 1997) comprises four items that measure global subjective happiness. Respondents indicate on a 7-point scale the extent to which each of the four items describes them. Lyubomirsky and Lepper (1997) reported Cronbach’s alpha values between 0.79 and 0.94 across 14 American and Russian samples consisting of high school and college students, as well as adult and retired community samples. Support for convergent and discriminant validity was also reported. In Africa, Agbo (2021) found that a one-factor model yielded adequate fit and sufficient reliability scores for Nigerian samples consisting of students and working populations. The scale had positive correlations with measures of well-being (e.g. satisfaction with life) and negative correlations with measures of ill-being (e.g. depression).

#### The Patient Health Questionnaire-9

The Patient Health Questionnaire-9 (PHQ-9; Kroenke et al., 2001) comprises nine items, and is a diagnostic tool used to assess the symptoms of depressive disorders on a scale with four response options, namely 0 (*not at all*), 1 (*several days*), 2 (*more than half of the days*) and 3 (*nearly every day*). Kroenke et al. (2001) reported support for criterion, construct and external validity with sufficient reliability scores in a patient sample in primary care (α = 0.86) and an obstetrics-gynaecology patient sample (α = 0.86), as well as test-retest reliability. Botha (2011) reported a one-factor confirmatory factor analysis (CFA) model with sufficient criterion-related validity and reliability (Cronbach’s α = 0.86) for the English version of the PHQ-9 in a multicultural South African sample. Appiah et al. (2020) found support for a 2-factor exploratory structural equation modelling model, with an ɷ-value of 0.76 for the Twi-translation of the scale in a rural adult Ghanaian sample.

#### The Patient Health Questionnaire-4

The Patient Health Questionnaire-4 (PHQ-4; Kroenke et al., 2009) is a very brief screening tool for symptoms of anxiety and depression (measured by two items each). Participants must indicate to what extent they have been bothered by these problems over the past 2 weeks. Response options vary from 0 (*not at all*) to 3 (*nearly every day*). Kroenke et al. (2009) reported the PHQ-4 to be a valid tool to screen for anxiety and depression. The PHQ-4 has further been validated in a German sample (Löwe et al., 2010) and used in several non-Western samples (e.g. Lenz & Li, 2022; Materu et al., 2020).

### Ethical considerations

Data were collected between 2014 and 2015 for Sample 1, in 2018 for Sample 2, between 2017 and 2019 for Sample 3, and in 2017 for Sample 4. All participants gave written informed consent and participated voluntarily in the study. Samples 1, 2 and 4 did not receive incentives for participation in the study, while Sample 3 received a small token of appreciation. All data were handled confidentially.

A research committee approach, using standard forward and back translation procedures, was employed to translate the original English version of the HILS into Setswana. Setswana is one of the 11 official languages that are spoken in South Africa, and is the main language spoken in the areas of South Africa where the data for Sample 3 were collected. Firstly, the scale was translated to Setswana by a bilingual speaker; secondly, the scale was back-translated into English by an independent translator; and then a research committee (consisting of academics with Setswana as first language and who were fluent in English, a professional translator, subject experts and members from the target communities) compared the back-translated English version with the original English version (Brislin, 1980; Van De Vijver & Humbleton, 1996; Van De Vijver & Leung, 1997).

For Samples 1 and 3, ethics approval was obtained from the Health Research Ethics Committee of the North-West University, South Africa, with ethics approval numbers: NWU 00002-07-A2 (Sample 1) and NWU-00016-10-A1 (Sample 3). In addition, ethics approval was obtained from the Department of Health of the North West Province for Sample 3. For Sample 2, ethics approval was obtained from the Faculty of Humanities Research Ethics Committee of the University of Johannesburg, ethics approval number: REC- 01-092-2017. For Sample 4, ethics approval was obtained from the University of Ghana Ethics Committee for Human Research, ethics approval number: ECH 086 16–17.

### Data analysis

The data were analysed in five stages.

#### Stage 1: Descriptive statistics of individual scale items

We used IBM^®^ Statistical Package for the Social Sciences (SPSS) 27 to calculate the mean, standard deviation, skewness and kurtosis for each item of the HILS across the four samples.

#### Stage 2: Factorial validity

The factor structure of the HILS was determined by applying confirmatory factor analysis (CFA) in Mplus 8.3 (Muthén & Muthén, 1998–2019). Full information likelihood estimation was used to handle missing data and the robust maximum likelihood estimator (MLR) was applied. The χ^2^-statistic, comparative fit index (CFI, Bentler, 1990), Tucker-Lewis index (TLI, Tucker & Lewis, 1973), root mean square error of approximation (RMSEA, Steiger & Lind, 1980) and the standardised root mean square residual (SRMR) were used to evaluate model fit. Models are deemed to display a good fit when the χ^2^-statistic has nonsignificant *p*-values (Byrne, 2012); the CFA and TLI values are larger than 0.95 (Byrne, 2012; Hu & Bentler, 1999); and RMSEA and SRMR values are less than 0.05 (values less than 0.08 indicate reasonable model fit; Byrne, 2012). The value of the χ^2^-statistic is sensitive to sample size (Byrne, 2012); therefore, the CFI, TLI, RMSEA and SRMR were primarily used to determine model fit.

#### Stage 3: Internal consistency reliability

The formula used by Sánchez-Oliva et al. (2017) was applied to calculate model-based omega coefficients of composite reliability.

#### Stage 4: Convergent and divergent validity

Pearson’s correlation coefficients were calculated in IBM^®^ SPSS Statistics 27 to determine convergent and divergent validity of the HILS. We also calculated the attenuation-corrected correlation coefficients by dividing the Pearson’s correlation coefficient by the square root of the product of the two (sub-)scales’ omega hierarchical coefficients of reliability to compensate for the lack of reliability of the scales (Borneman, 2010). Although the Pearson’s *r*-values are also reported, our interpretation of the correlations between the HILS and the criterion scales were based upon the attenuation-corrected *r*-values. This is because the relationships between constructs are attenuated by random measurement error (Borneman, 2010). When this attenuation is corrected, the relationships between the scales are estimated as if they were free from random error, thus estimating the true relationship between the HILS and the criterion scales (see Borneman, 2010).

#### Stage 5: Measurement invariance

Invariance of the HILS across the different samples was investigated in a hierarchical series of steps using Mplus 8.3 (Muthén & Muthén, 1998–2019). For configural invariance, the number of factors, as well as the structure of fixed and freely estimated parameters were assumed to be similar across the groups, but no equality constraints were applied (Byrne, 2012). For metric invariance, the factor loadings were constrained to be equal for all the groups, and for scalar invariance equality constraints were applied to factor loadings and intercepts. If configural invariance had not been supported, the measures were deemed noninvariant. However, if configural invariance had been supported but either metric or scalar invariance not, nonequivalant factor loadings or intercepts, respectively, were freed one at a time to look for partial metric or partial scalar invariance (Putnick & Bornstein, 2017). High MIs and EPCs were used to determine which parameters had to be freely estimated to yield partial metric or partial scalar invariance (Byrne, 2012). Differences smaller than 0.01 in the CFI values and smaller than 0.015 in the RMSEA values of the nested models, indicate measurement invariance. We report the likelihood ratio test but did not use it for decision-making, because of its sensitivity to sample size (Chen, 2007; Cheung & Rensvold, 2002).

## Results

### Stage 1: Descriptive statistics of the individual scale items of the Harmony in Life Scale for Samples 1–4

The means, standard deviations, skewness and kurtosis values for the individual scale items of the HILS for all samples are presented in Table 1-A1. Mean values were between 4.73 (standard deviation [SD] = 1.629; item 3) and 5.36 (SD = 1.404, item 4) for Sample 1; between 4.98 (SD = 1.530, item 2) and 5.44 (SD = 1.310, item 5) for Sample 2; between 5.43 (SD = 1.449, item 2) and 6.03 (SD = 1.371, item 3) for Sample 3; and between 5.03 (SD = 1.449, item 4) and 5.51 (SD = 1.335, item 1) for Sample 4.

Skewness values were between -0.690 (item 3) and -1.310 (item 4) for Sample 1; between -1.239 (item 4) and -0.775 (item 2) for Sample 2; between -1.833 (item 3) and -1.106 (item 2) for Sample 3; and between -0.966 (item 1) and -0.652 (item 2) for Sample 4. Kurtosis values were between -0.362 (item 3) and 1.553 (item 4) for Sample 1; between -0.186 (item 2) and 1.432 (item 4) for Sample 2; between 0.804 (item 2) and 3.243 (item 3) for Sample 3; and between 0.256 (item 3) and 1.035 (item 1) for Sample 4. Except for exceptions in Sample 3, there were no significant deviations from normality as indicated by skewness and kurtosis values that were smaller than two in absolute value (Bandalos & Finney, 2010).

### Stage 2: Factorial validity of the Harmony in Life Scale

Confirmatory factor analysis was used to determine the factor structure of the HILS. The fit indices for the HILS in the various samples are presented in Table 2. Except for Sample 3, all CFI values exceeded 0.95, TLI values were close to 0.95, RMSEA values were smaller than 0.08 in most instances, while the SRMR values were smaller than 0.05. The HILS therefore showed acceptable fit for all samples, except for Sample 3.

**TABLE 2:** Global fit indices for the Harmony in Life Scale in the various samples.

Latent model	χ^2^	*df*	*p*	CFI	TLI	RMSEA	90% CI	SRMR
Sample 1 (Multicultural, SA1)	10.592	5	0.0601	0.985	0.970	0.063	0.000; 0.115	0.029
Sample 2 (Multicultural, SA2)	19.546	5	0.0015	0.972	0.943	0.083	0.046; 0.123	0.039
Sample 3 (Setswana, SA)	52.842	5	< 0.01	0.890	0.779	0.155	0.119; 0.194	0.044
Sample 4 (Ghana)	12.013	5	0.0346	0.971	0.943	0.058	0.014; 0.101	0.038

χ^2^, Chi-square test statistic; *df*, degrees of freedom; *p*, probability value of chi-square test; CFI, comparative fit index; TLI, Tucker-Lewis index; RMSEA, root mean square error of approximation; CI, confidence interval of the RMSEA; SRMR, standardised root-mean square residual; SA, South African; SA1, South African Sample 1; SA2, South African Sample 2.

Apart from the global fit indices, item-level parameters, namely the standardised factor loadings, the items’ residual variances and the items’ *R*^2^-values, were also considered (see Table 3). The standardised factor loadings ranged between 0.545 and 0.939 in the various samples, thereby supporting the factorial validity of the HILS. Although global model fit was insufficient for Sample 3, the factor loadings ranged between 0.701 and 0.819. For the two multicultural South African samples (Samples 1 and 2), the factor loadings and *R*^2^-values of items 1, 2 and 3 were notably higher than the factor loadings and *R*^2^-values of items 4 and 5. This aligns with the findings by Kjell et al. (2016) where the first three items of the HILS showed the highest item-total correlations. The first three items also showed the highest factor loadings on the harmony factor when a two-factor model was applied to combined HILS and SWLS data (Kjell et al., 2016; Kjell & Diener, 2021). When abbreviating the HILS, Kjell and Diener (2021) proposed that the first three items of the HILS (containing the words ‘harmony’ and ‘balance’) are most suitable for an abbreviated scale as these items refer to the most central aspects of harmony in life, compared to the last two items that refer to ‘accept’ (item 4) and ‘fitting in’ (item 5). The same pattern was not observed for Samples 3 and 4. Factor loadings were relatively close to each other for Samples 3 and 4, except for item 4 that showed a substantially smaller factor loading for Sample 4 compared to the other items. The residual variances for Samples 1 and 2 were also smaller than for Samples 3 and 4. Because Sample 3 did not produce a good fitting baseline model, convergent and divergent validity as well as measurement invariance were only investigated for Samples 1, 2 and 4.

**TABLE 3:** Standardised factor loadings, residual variances, *R*^2^-values and omega coefficients of the Harmony in Life Scale for the various samples.

Item	Sample 1 Multicultural, SA1			Sample 2 Multicultural, SA2			Sample 3 Setswana, SA			Sample 4 Ghana		
	FL	ResVar	*R* ^2^	FL	ResVar	*R* ^2^	FL	ResVar	*R* ^2^	FL	ResVar	*R* ^2^
1	0.852	0.274	0.726	0.835	0.303	0.697	0.814	0.338	0.662	0.804	0.353	0.647
2	0.861	0.259	0.741	0.895	0.198	0.802	0.705	0.503	0.497	0.680	0.538	0.462
3	0.905	0.181	0.819	0.939	0.118	0.882	0.819	0.329	0.671	0.815	0.336	0.664
4	0.699	0.512	0.488	0.640	0.591	0.409	0.772	0.404	0.596	0.545	0.703	0.297
5	0.654	0.572	0.428	0.658	0.556	0.434	0.701	0.508	0.492	0.741	0.451	0.549
Omega coefficient	0.90	-	-	0.90	-	-	0.87	-	-	0.84	-	-

FL, factor loading; ResVar, residual variance; *R*^2^, proportion of variance explained by the factor; SA, South African; SA1, South African Sample 1; SA2, South African Sample 2.

### Stage 3: Internal consistency reliability

The HILS showed sufficient reliability scores for all samples. The model-based omega coefficient of composite reliability ranged between 0.84 and 0.90 (see Table 3).

### Stage 4: Convergent and divergent validity

Different scales were included in the research battery used for each of the samples, hence the criterion scales differed across samples. All criterion scales were screened for factorial validity (Table 4) and reliability (see Table 5). Only (sub)scales that showed sufficient model fit and reliability scores were included in the analysis. Note that item 9 of the MLQ_P was removed for the Ghanaian sample (Sample 4) as this negatively worded item posed problems with model fit.

**TABLE 4:** Global fit indices for the criterion-scales used in the various samples.

Latent model	χ^2^	*df*	*p*	CFI	TLI	RMSEA	90% CI	SRMR
**Sample 1 (Multicultural, SA1)**								
MHC_EWB	0.000	0	< 0.01	1.000	1.000	0.000	0.000; 0.000	0.000
MHC_PWB	21.195	9	0.0118	0.976	0.959	0.069	0.031; 0.107	0.029
MLQ_P	3.966	5	0.5544	1.000	1.006	0.000	0.000; 0.073	0.015
MLQ_S	8.488	5	0.1313	0.992	0.984	0.049	0.000; 0.105	0.018
AFM_PA	155.253	35	< 0.01	0.950	0.935	0.109	0.092; 0.127	1.032†
AFM_NA	108.023	35	< 0.01	0.968	0.959	0.085	0.067; 0.104	0.822†
PHQ-9	98.800	27	< 0.01	0.974	0.966	0.096	0.076; 0.117	0.967†
**Sample 2 (Multicultural, SA2)**								
SWLS	15.951	5	< 0.01	0.981	0.962	0.072	0.034; 0.112	0.021
MLQ_P	26.927	5	< 0.01	0.965	0.930	0.101	0.066; 0.140	0.019
SPANE_PE	21.369	9	0.0111	0.985	0.975	0.057	0.026; 0.088	0.019
SPANE_NE	43.180	9	< 0.01	0.960	0.933	0.094	0.067; 0.123	0.036
SHS	1.545	2	0.4617	1.000	1.003	0.000	0.000; 0.089	0.008
PHQ-4	63.337	2	< 0.01	0.979	0.938	0.268	0.214; 0.327	1.360†
**Sample 4 (Ghana)**								
SWLS	16.626	5	< 0.01	0.969	0.939	0.075	0.037; 0.116	0.030
MLQ_P (item 9 removed)	5.592	2	0.0611	0.980	0.939	0.066	0.000; 0.133	0.027
PANAS_NA	115.838	35	< 0.01	0.921	0.898	0.075	0.060; 0.090	0.046

MHC_EWB, Mental Health Continuum (Emotional Well-being subscale); MHC_PWB, Mental Health Continuum (Psychological Well-being subscale); MLQ_P, Meaning in Life Questionnaire (Presence subscale); MLQ_S, Meaning in Life Questionnaire (Search subscale); AFM_PA, Affectometer (Positive Affect subscale); AFM_NA, Affectometer (Negative Affect subscale); PHQ-9, Patient Health Questionnaire-9; SWLS, Satisfaction with Life Scale; SPANE_PE, Scale of Positive and Negative Experiences (Positive Experiences subscale); SPANE_NE, Scale of Positive and Negative Experiences (Negative Experiences subscale); SHS, Subjective Happiness Scale; PHQ-4, Patient Health Questionnaire-4; PANAS_NA, The Positive Affect and Negative Affect Schedule (Negative Affect subscale); χ^2^, Chi-square test statistic; *df*, degrees of freedom; *p*, probability value of chi-square test; CFI, comparative fit index; TLI, Tucker-Lewis index; RMSEA, root mean square error of approximation; CI, confidence interval of the RMSEA; SRMR, standardised root-mean square residual; SA1, South African Sample 1; SA2, South African Sample 2.

† , weighted root mean square residual.

**TABLE 5:** Correlations between the Harmony in Life Scale and other measures of well-being and ill-being for Samples 1, 2 and 4.

Criterion scales per sample	Omega coefficient of criterion (sub-scale)	Pearson’s *r*-value†	Attenuation corrected Pearson’s *r*-value†
**Sample 1, Multicultural, SA1**			
MHC_EWB	0.83	0.601*	0.695
MHC_PWB	0.88	0.623*	0.700
MLQ_P	0.89	0.670*	0.749
MLQ_S	0.90	−0.183*	−0.203
AFM_PA	0.90	0.716*	0.796
AFM_NA	0.91	−0.596*	−0.659
PHQ-9	0.93	−0.538*	−0.588
**Sample 2, Multicultural, SA2**			
SWLS	0.88	0.756*	0.849
MLQ_P	0.92	0.644*	0.708
SPANE_PE	0.90	0.643*	0.714
SPANE_NE	0.87	−0.471*	−0.532
SHS	0.86	0.612*	0.696
PHQ-4	0.91	−0.503*	−0.556
**Sample 4, Ghana**			
SWLS	0.85	0.582*	0.689
MLQ_P (item 9 removed)	0.78	0.570*	0.704
PANAS_NA	0.88	−0.308*	−0.358

HILS, Harmony in Life Scale; MHC_EWB, Mental Health Continuum (Emotional Well-being subscale); MHC_PWB, Mental Health Continuum (Psychological Well-being subscale); MLQ_P, Meaning in Life Questionnaire (Presence subscale); MLQ_S, Meaning in Life Questionnaire (Search subscale); AFM_PA, Affectometer (Positive Affect subscale); AFM_NA, Affectometer (Negative Affect subscale); PHQ-9, Patient Health Questionnaire-9; SWLS, Satisfaction with Life Scale; SPANE_PE, Scale of Positive and Negative Experiences (Positive Experiences subscale); SPANE_NE, Scale of Positive and Negative Experiences (Negative Experiences subscale); SHS, Subjective Happiness Scale; PHQ-4, Patient Health Questionnaire-4; PANAS_NA, The Positive Affect and Negative Affect Schedule (Negative Affect subscale); SA1, South African Sample 1; SA2, South African Sample 2.

* , *p* < 0.01;

† , Value represents the correlation between the HILS and the relevant criterion scales.

The model-based omega coefficients of composite reliability of each criterion scale, Pearson’s *r*-values, and the attenuation-corrected *r*-values are presented in Table 5. The HILS showed strong positive correlations with measures of well-being (e.g. positive mental health, meaning, positive affect/experiences and happiness), medium to strong negative correlations with measures of negative affect and ill-being (e.g. negative affect/experiences and depression), and a weak negative correlation with search for meaning. These findings point towards the convergent and divergent validity of the HILS.

### Stage 5: Measurement invariance

We wanted to test whether the English version of the HILS was invariant for Samples 1, 2 and 4. However, because we expected that cultural differences could influence the results, we first tested for invariance between the multicultural South African samples (Samples 1 and 2), whereafter we also included the Ghanaian sample (Sample 4) in the analysis. The results for the measurement invariance of the HILS are presented in Table 6.

**TABLE 6:** Measurement invariance of the Harmony in Life Scale for Samples 1 and 2, and for Samples 1, 2 and 4.

Model	χ^2^	*df*	*p*	CFI	RMSEA	Model comparison	∆χ^2^	*df*	*p*	∆ CFI	∆ RMSEA
Samples 1 and 2											
Invariance Model 1	31.078	10	< 0.001	0.976	0.077	-	-	-	-	-	-
Invariance Model 2	37.234	14	< 0.001	0.974	0.068	2 vs 1	3.877	4	0.422	−0.002	−0.009
Invariance Model 3	45.172	18	< 0.001	0.969	0.065	3 vs 2	5.793	4	0.215	−0.005	−0.003
Samples 1, 2 and 4											
Invariance Model 1	42.041	15	< 0.001	0.975	0.069	-	-	-	-	-	-
Invariance Model 2A	74.542	23	< 0.001	0.953	0.077	2A vs 1	38.314	8	< 0.001	−0.022	0.008
Invariance Model 2B	56.209	21	< 0.001	0.968	0.067	2B vs 1	12.710	6	0.047	−0.007	−0.002
Invariance Model 3A	127.856	29	< 0.001	0.910	0.095	3A vs 2B	122.989	8	< 0.001	−0.058	0.028
Invariance Model 3B	98.511	27	< 0.001	0.935	0.084	3B vs 2B	75.428	6	< 0.001	−0.033	0.017
Invariance Model 3C	69.497	25	< 0.001	0.960	0.069	3C vs 2B	16.010	4	0.003	−0.008	0.002

Note: Samples 1 and 2: Invariance Model 1, configural invariance model; Invariance Model 2, metric invariance model; Invariance Model 3, scalar invariance model; Samples 1, 2 and 4: Invariance Model 1, configural invariance model; Invariance Model 2A, metric invariance model; Invariance Model 2B, partial metric invariance model with the factor loading of item 5 freely estimated for all groups; Invariance Model 3A, partial scalar invariance model with the factor loading of item 5 freely estimated for all groups; Invariance Model 3B, partial scalar invariance model with the factor loading of item 5 and the intercept of item 4 factor freely estimated for all groups; Invariance Model 3C, partial scalar invariance model with the factor loading of item 5 and the intercepts of items 4 and 5 freely estimated for all groups.

χ^2^, Chi-square test statistic; *df*, degrees of freedom; *p*, probability value of chi-square test; CFI, comparative fit index; RMSEA, root mean square error of approximation; CI, confidence interval of the RMSEA.

Full scalar invariance was supported for the two multicultural South African samples (Samples 1 and 2) as indicated by ǀ∆CFIǀ values smaller than 0.01 and ǀ∆RMSEAǀ values smaller than 0.015. Only partial scalar invariance was supported when the Ghanaian sample (Sample 4) was also included in the analysis. Specifically, the configural model (Invariance Model 1) yielded adequate fit for the data. A ǀ∆CFIǀ value larger than 0.01 indicated insufficient metric invariance (Invariance Model 2A). The factor loading of item 5 had a high modification index (MI = 14.545) for the Ghanaian group and the factor loading was allowed free estimation for all groups. This model (Invariance Model 2B) yielded a ǀ∆CFIǀ value smaller than 0.01 and a ǀ∆RMSEAǀ value smaller than 0.015, indicating support for partial metric invariance. Partial scalar invariance (Invariance Model 3C), as indicated by a ǀ∆CFIǀ value smaller than 0.01 and a ǀ∆RMSEAǀ value smaller than 0.015 after the intercepts of items 4 (MI = 24.843 for the Ghanaian sample; Invariance Model 3A) and 5 (MI = 24.172 for the Ghanaian sample, Invariance Model 3B) were allowed free estimation, one at a time, for all groups.

## Discussion

The aim of this study was to evaluate the psychometric properties and measurement invariance of the HILS for different South African and Ghanaian samples. A single-factor solution fitted all samples, except for Sample 3 (the South African sample who completed the scale in Setswana). The HILS showed sufficient reliability with ɷ-values larger than 0.80. Convergent and divergent validity were supported for Samples 1 (a multicultural South African sample completing the scales in English), 2 (a multicultural South African sample completing the scales in English) and 4 (a Ghanaian sample completing the scales in English). Full scalar invariance was supported for Samples 1 and 2, but only partial scalar invariance when Sample 4 was added to the analysis. Some findings will be discussed.

### Good model fit across the different African samples

The HILS displayed good fit across different samples from Africa who completed the questionnaire in English. Because the samples in this study may be very different in terms of cultural orientation compared to the samples for which the validity of the HILS was originally investigated by Kjell et al. (2016), this is very significant and suggests that the scale may be useful across different and diverse groups. It may also confirm the importance and prominence of harmony as a facet of psychosocial well-being across different groups.

### Findings for the Setswana-speaking South African sample

The HILS did not fit well for the Setswana-speaking sample (Sample 3). It may be that the exact meaning of some scale items was altered when the HILS was translated from English to Setswana. Specifically, in the Setswana language there is not a separate word for ‘harmony’. Harmony, which appears in items 1 and 3 of the HILS, has been translated with the Setswana word ‘kagiso’ which means ‘peace’, and can refer to being neighbourly or getting along with others, rather than inner harmony which may be the connotation with the English word in the context of the scale items’ phrasing (see Metz, 2017). A word with similar connotation does not exist in Setswana. This difference in nuance may have affected the way in which participants interpreted items 1 and 3 of the Setswana version of the scale, implying that this translation may measure a related but different construct.

Another possible explanation for the finding may be that the HILS does not capture harmony as it is understood in the Batswana cultural context. As indicated earlier, harmony is understood in terms of social relationships in African contexts (see Metz, 2017, for a discussion), and the scale items of the HILS relate more to inner harmony (items 1 to 3) and the external environment (items 4 and 5). Therefore, completely different scale items may need to be developed to capture the cultural understanding and meaning of harmony.

### Convergent and divergent validity of the Harmony in Life Scale

The HILS showed acceptable convergent and divergent validity for Samples 1, 2 and 4. Specifically, we found strong positive correlations between the HILS and measures of well-being (e.g. positive mental health, meaning, positive affect, positive experiences, and happiness), and medium to strong negative correlations between the HILS and measures of ill-being (e.g. negative affect, negative experiences, and depression) and a weak negative relationship with search for meaning in life. These findings are in line with the findings of Kjell et al. (2016) and Satici and Tekin (2017) discussed above, where the HILS correlated positively with measures of well-being and negatively with measures of mental ill-being.

### Measurement invariance

Full scalar invariance was supported for Samples 1 and 2, while partial scalar invariance was supported for Samples 1, 2 and 4. The different groups can therefore be compared in terms of the estimated latent mean scores obtained for the HILS. Interestingly, the factor loading of item 4 (that refers to accepting one’s life conditions) and the intercepts of items 4 and 5 (that refers to fitting in with one’s surroundings) were noninvariant for the Ghanaian group. These two items seem to refer more to external conditions, while the first three items (item 1 [referring to a harmonious lifestyle]; item 2 [referring to overall balance in life]; item 3 [referring to being in harmony]) seem to refer more to what is within a person’s personal sphere of influence. The first three items, that can be considered to be more central to harmony in life (Kjell & Diener, 2021), may be more invariant than items 4 and 5 which are more externally focused.

## Limitations and recommendations

Despite the contribution of this study, there are also limitations. Firstly, while the current study is the first to evaluate performance of the HILS in African countries, only two countries were selected, which do not represent the full African population. Furthermore, the samples within the countries were not representative of the countries’ populations. In terms of ethical principles such as fair selection and scientific validity, we acknowledge the limitations of the study, and do not suggest that the findings are generalisable to the populations within the respective countries, or to the wider African population. It will be worthwhile to study harmony as a construct and the measurement thereof in more African countries in future, using representative samples. Secondly, although the English version of the HILS shows potential for use in the multicultural South African and Ghanaian samples, this study only provides preliminary support for its use and future research may explore whether these results replicate in other samples. Specifically, future research can explore the psychometric properties of the HILS from a cultural perspective and, should the scale be translated into different African and global languages, specifically attend to the semantical and cultural meaning of constructs. Lastly, although our sample sizes were adequate according to minimum sample size guidelines for performing factor analysis, future research may use larger sample sizes to account for challenges associated with smaller sample sizes, such as biased standard errors and questionable quality of the fit statistics (see Kyriazos, 2018 in this regard).

## Conclusion

The HILS shows potential for use in the current samples, except for the Setswana-speaking South African sample. One should also take cognisance thereof that the HILS may measure a different, but related construct to harmony in the Setswana speaking sample (who completed the Setswana translation of the HILS); alternatively, that the HILS does not capture the cultural understanding and meaning of harmony in a Batswana cultural context. The findings emphasise the importance of language, and how different notions may be expressed in different languages, considering that words with exactly the same meaning may not exist in different languages. Cultural meanings and understandings are expressed in language, and nuances may differ in different languages. This stresses the importance of cultural and/or contextual and linguistic differences and how these impact the measurement of psychological constructs. In this regard, future research should qualitatively explore the nuances and manifestations of harmony in various African and other global contexts. New measures, that capture these meanings in the local languages, can then be developed.

## Appendix 1

**TABLE 1-A1:** Descriptive Statistics of the Individual Scale Items of the HILS for Samples 1–4.

Item	Sample 1: Multicultural, SA1				Sample 2: Multicultural, SA2				Sample 3: Setswana, SA				Sample 4: Ghana			
	M	SD	Skewness	Kurtosis	M	SD	Skewness	Kurtosis	M	SD	Skewness	Kurtosis	M	SD	Skewness	Kurtosis
1	4.99	1.577	−0.797	−0.184	5.20	1.425	−0.863	0.265	5.84	1.409	−1.644	2.547	5.51	1.335	−0.966	1.035
2	4.88	1.553	−0.796	−0.251	4.98	1.530	−0.775	−0.186	5.43	1.449	−1.106	0.804	5.10	1.321	−0.652	0.517
3	4.73	1.629	−0.690	−0.362	5.00	1.540	−0.783	−0.133	6.03	1.371	−1.833	3.243	5.31	1.359	−0.667	0.256
4	5.36	1.404	−1.310	1.553	5.40	1.354	−1.239	1.432	5.80	1.394	−1.574	2.243	5.03	1.449	−0.799	0.542
5	5.31	1.368	−1.164	1.257	5.44	1.310	−1.150	1.066	5.75	1.440	−1.526	2.033	5.24	1.448	−0.896	0.711

HILS, Harmony in Life Scale; M, mean; SD, standard deviation; SA, South African; SA1, South African Sample 1; SA2, South African Sample 2.
